# Overweight, physical activity, tobacco and alcohol consumption in a cross-sectional random sample of German adults

**DOI:** 10.1186/1471-2458-6-233

**Published:** 2006-09-18

**Authors:** Mark M Haenle, Stefan O Brockmann, Martina Kron, Ursula Bertling, Richard A Mason, Gerald Steinbach, Bernhard O Boehm, Wolfgang Koenig, Peter Kern, Isolde Piechotowski, Wolfgang Kratzer

**Affiliations:** 1 Department of Internal Medicine I, University Hospital Ulm, Robert-Koch-Str. 8, 89081 Ulm, Germany; 2 Baden-Württemberg State Health Office, District Government Stuttgart, Germany; 3 Department of Biometry and Medical Documentation, University of Ulm, Ulm, Germany; 4 Local Health Department Ravensburg, Leutkirch, Germany; 5 Central Department Clinical Chemistry, University Hospital Ulm, Ulm, Germany; 6 Department of Internal Medicine II, University Hospital Ulm, University of Ulm, Ulm, Germany

## Abstract

**Background:**

There is a current paucity of data on the health behaviour of non-selected populations in Central Europe. Data on health behaviour were collected as part of the EMIL study which investigated the prevalence of infection with *Echinococcus multilocularis *and other medical conditions in an urban German population.

**Methods:**

Participating in the present study were 2,187 adults (1,138 females [52.0%]; 1,049 males [48.0%], age: 18–65 years) taken from a sample of 4,000 persons randomly chosen from an urban population. Data on health behaviour like physical activity, tobacco and alcohol consumption were obtained by means of a questionnaire, documentation of anthropometric data, abdominal ultrasound and blood specimens for assessment of chemical parameters.

**Results:**

The overall rate of participation was 62.8%. Of these, 50.3% of the adults were overweight or obese. The proportion of active tobacco smokers stood at 30.1%. Of those surveyed 38.9% did not participate in any physical activity. Less than 2 hours of leisure time physical activity per week was associated with female sex, higher BMI (Body Mass Index), smoking and no alcohol consumption. Participants consumed on average 12 grams of alcohol per day. Total cholesterol was in 62.0% (>5.2 mmol/l) and triglycerides were elevated in 20.5% (≥ 2.3 mmol/l) of subjects studied. Hepatic steatosis was identified in 27.4% of subjects and showed an association with male sex, higher BMI, higher age, higher total blood cholesterol, lower HDL, higher triglycerides and higher ALT.

**Conclusion:**

This random sample of German urban adults was characterised by a high prevalence of overweight and obesity. This and the pattern of alcohol consumption, smoking and physical activity can be considered to put this group at high risk for associated morbidity and underscore the urgent need for preventive measures aimed at reducing the significantly increased health risk.

## Background

The findings of current epidemiological research show, that a few largely preventable risk factors account for most of the world's disease burden. Chronic diseases – including cardiovascular conditions, obesity, diabetes, stroke, cancers and respiratory diseases – account for 59% of the 57 million deaths annually, and 46% of the global disease burden [1]. This reflects a significant change in diet habits and physical activity levels worldwide as a result of industrialization, urbanization, economic development and increased food market globalization [1].

These changes have immense importance, both in terms of economics and in healthcare administration [2,3]. Of these, obesity is the fastest growing health-related problem worldwide [4,5]. Obesity, diabetes mellitus and hypercholesterolemia, all of which are associated with lifestyles characterized by unbalanced diets high in calories and with inadequate physical activity, are considered risk factors for many diseases [6]. Increased consumption of alcohol and tobacco products also constitutes significant sources of excess morbidity and mortality in the population [7]. Studies by the National center for chronic Disease Prevention and Health Promotion in the USA have shown that, depending on region, the impact of the risk factors overweight, inadequate physical activity, tobacco and alcohol may vary by up to 20% between populations and regions [8]. Similar findings were published by Meyer et al. showing significant regional differences in the prevalence of high-risk alcohol consumption within Germany [9]. To date, only few prospective studies have combined extensive phenotyping and medical histories to define population-based risk profiles. Moreover, there is a paucity of data acquired from locally defined population samples, especially urban populations [10,11].

Based on an initiative of the state government of Baden-Württemberg, which recognised the importance of evaluating the danger of *E. multilocularis *infection in an urban population in southern Germany where the high rate of infestation with *Echinococcus multilocularis *among the fox population has become a recognised regional public health issue [12], and because of the increasing importance of lifestyle-associated diseases, the State Health Department of Baden-Württemberg and the University of Ulm have cooperated to design and initiate the **E**chinococcus **M**ultilocularis and **I**nternal Diseases in **L**eutkirch (EMIL) study. The original aim of assessing the prevalence of E. multilocularis infection was amended by additional aims in the field of public health and internal medicine.

Objective of the present study is to characterise the alcohol and tobacco consumption, physical activity, serum lipoproteins, liver enzymes, fatty liver and weight and height of an urban population sample by use of clinical chemistry, anthropometry, medical history and ultrasound examination and to assess the effect of these factors on the occurrence of steatosis hepatis, the amount of physical activity, the level of Gamma-glutamyl transpeptidase and on the level of total blood cholesterol.

The data are intended to serve as a basis for recommendations of public health authorities in Baden-Württemberg, in particular in formulating specific regional preventive measures [13].

## Methods

### Population

We conducted a population-based, cross-sectional health survey in the city of Leutkirch in southwestern Germany. 4,000 of 12,475 inhabitants of an urban population were randomly selected by the staff of the municipal registration office from the registry of inhabitants and invited to participate in the study. Of the 4000 randomly selected and invited participants, 107 were ineligible because of having moved from the area without forwarding address, resulting in a sample of 3893 subjects. A total of 2,445 persons aged 10–65 years participated in the study leading to a sample with a participation rate of 62.8%. Systematic investigation of non-responders by a short questionnaire showed that being away for the whole field period (47.4 %; 180 of 380 returned questionnaires) was the main reason for non response. Other reasons were acute (6.1%; 23 of 380) or chronic illness (4.5%; 17 of 380). Refusal by reason of lacking interest in the study or lack of time was reported by 38.7% (151 of 380). The remaining non-responders gave a disbelief in data security of their personal data or a doctor's recommendation not to take part in the study as reasons for non response. The following analysis considers only data derived from 2,187 adult subjects between 18 and 65 years. The survey was conducted from November 4^th ^to December 7^th^, 2002. All selected participants received an invitation by mail two months prior to the beginning of the field study. The letter included general information, aims and scope of the study as well as time and location of the appointment. A reminder was sent a week before the appointment. Each participant had the option of changing the date and time of his appointment by phoning a call-centre open 12 hours per day. If an invited person did not present for the appointment, he or she was called by phone (if available, or sent a new reminder via mail) offering a new appointment. Selected subjects not attending were asked for their reasons (standardized questionnaire) and were offered a second appointment. Those that did not show up were considered as non-participant.

### Public relations

The local press reported on the project some weeks prior to and several times during the field study. All general medical practitioners in the city received an information letter, explaining the design and purpose of the study, in case their respective patients contacted them. Two weeks before the field study started, three informational meetings were held to offer participants a forum for gaining more information about the study.

### Analytical and diagnostic techniques and equipment

Apart from the information gained by the use of questionnaires, the investigative techniques used in the study included methods from the field of anthropometry and clinical chemistry. Abdominal ultrasonography was used as a further diagnostic tool.

### Interview and questionnaire

Each interview was conducted by a trained interviewer. In order to reduce interviewer bias as much as possible, each interviewer underwent in-depth training by an interviewing specialist of the state health office. The standardized questionnaire included personal data (i.e. date of birth, gender, education, current work, diseases), health and social behaviour (i.e. sports activities, nutrition, alcohol consumption), as well as assumptive risk factors for infection with *E. multilocularis *(i.e. pets, forest and garden work or leisure activities) and other risk factors for medical diseases (i.e. smoking), previous medical records of participants and families and previous pharmaceutical records of the participants. In order to validate the results multiple crosschecked questions on the same topic were addressed to the participants. The interview was partially based on validated instruments from other, predominantly cardiovascular, studies (i. e. alcohol questions from the MONICA Study) [11,14].

For estimation of alcohol consumption, each subject was asked how much beer, wine, and spirits he or she had drunk on the previous workday and over the last weekend. Total alcohol intake was calculated by multiplying weekday consumption by five and adding this figure to weekend consumption. An average amount of alcohol intake in grams per day was derived. This recall method was validated in a subsample of 899 male participants of the first MONICA Augsburg survey in 1984/85 [14].

Physical activity was addressed by asking for leisure time physical activity (no activity, less than one hour per week, 1 to 2 hours per week and more than 2 hours per week) at the beginning of the questionnaire and later on in the questionnaire by a more extensive text block referring to workplace and leisure time physical activity in detail. Participants were asked how many hours of sports per week and in the following questions how many hours per week of outdoor sports (divided into summer and winter time) they perform. Further questions included the hours per week of physical activity at work and at home as well as the question of how many hours per week the participants' activity was enough "to break a sweat". Finally the participants were asked to rate their actual fitness on a scale from 1 to 10.

Smoking habits were assessed by asking if the participant was currently smoking, has never smoked or is an ex-smoker. Current and ex-smokers were asked for the number of cigarettes (in classes of 10) per day they are/were smoking. They also were asked for how many years they are/were smoking (less than 1 year, 1 to 3 years, more than 3 to five years, more than 5 to 10 years, more than 10 to 20 years and more than 20 years).

The participants were asked how often they consume sugary drinks or sweets (seldom or never, several times per month, several times per week, every day, several times per day). Vegetarian diet was addressed by asking the participants for how long they restricted themselves to this kind of diet (less than 1 year, 1 to 5 years or more than 5 years).

### Anthropometry

Based on recommendations of the WHO [15], we determined subjects' height, body weight, hip and waist circumference. Underweight, normal weight, overweight and obese were defined as follows: underweight = BMI <18.5 kg/m^2^; normal weight = BMI 18.5–<25 kg/m^2^; overweight = BMI 25–<30 kg/m^2^; obesity, grade I = BMI 30–<35 kg/m^2^; obesity, grade II = BMI 35–<40 kg/m^2^; obesity, grade III = BMI = 40 kg/m^2^.

### Laboratory tests and procedures

About 25 ml of venous blood was obtained from a cubital vein in a maximum of three attempts. Laboratory testing included analytical clinical chemistry for liver enzymes, lipids and other biochemical values (serum or plasma levels of triglycerides, cholesterol, gamma-glutamyl transpeptidase (GGT), aspartate aminotransferase (AST) and alanine aminotransferase (ALT) were determined using the Dimension XL (Dade Behring Inc., Newark, DE, USA)). The laboratory studies described in this section were conducted in the laboratory of the University Hospital Ulm, which routinely provides services for clinical studies. As far as possible, tests were conducted according to the guidelines of the IFCC (International Federation of Clinical Chemistry and Laboratory Medicine). Above 5.2 mmol/l total cholesterol levels were considered elevated. Triglycerides were defined as elevated at 2.3 mmol/l or above this cut-off value.

### Ultrasonography

Four identical HDI 5000 units (ATL Ultrasound, Philips Medical Systems, Bothell, WA, USA) were used. Probe settings were specifically developed and standardized for the study and were defined for all four units. Settings were checked at least once each day the scanners were in operation. In their invitations, study participants were requested to abstain from oral intake of food and fluids for at least 4 hours prior to the appointment and were asked not to smoke. Prior to the examination, subjects' last oral intake was documented. Subjects then underwent examination of the liver, gallbladder, biliary tract, spleen and both kidneys. All ultrasound examinations were performed by examiners specially trained for the diagnostic protocol used in the study. All relevant pathological findings were presented to an experienced supervisor (> 3000 ultrasound examinations per year) and documented using a standardized documentation instrument. In addition, relevant findings were captured on videotape and print-outs. For each participant, short sequences of the actual examination together with short sequences acquired from two ultrasound phantoms (Model 411 LE 0,5, Gammex RMI, Middleton, WI, USA) were transferred to a corresponding software program (HDI Lab, SW Version 1.91c, ATL Ultrasound, Philips, Bothell, WA, USA).

### Statistical methods

Absolute and relative frequencies were calculated for qualitative factors in the descriptive-statistical analysis, while, for quantitative factors, mean and standard deviation, as well as median, minimum and maximum were calculated.

Multiple logistic regression was performed in order to identify risk factors for the development of steatosis hepatis and to show potential association between physical activity and other factors. Results of logistic regression are presented as Odds Ratios (OR) together with 95% confidence intervals (CI) and corresponding p values for all factors which were considered in the model. For the laboratory parameters GGT and total cholesterol, analysis of covariance was used to assess the effect of diverse factors. Results are presented as parameter estimates, corresponding 95% CI and p-values for all factors which were considered in the model. Statistical analysis was performed using SAS 8.02 (SAS, Heidelberg, Germany).

### Participant result information

Personal data were strictly segregated from the results of the individual examinations. Personal data were archived in the Local Health Department and were only released to medical personnel of the University Hospital Ulm upon written permission from the subject. The study results were archived anonymously at the University of Ulm. Participants with pathologic findings were addressed personally or through their general practitioner as soon as possible, while personal data remained undisclosed to all persons outside the Local Health Department.

### Ethical agreement and informed consent

The study meets the international agreements of the revised version of the World Medical Association Declaration of Helsinki about ethical principles for medical research involving human subjects in the version from 2000 and was approved by the ethics committee of the State Medical Chamber Baden-Württemberg. A written consent was obtained from each participant of the study.

## Results

### Study participation/Study population

Of the 3,893 randomly invited residents, 2,187 of the adult population participated (1,138 females [52.0%], average age 42.5 ± 12.9 years; 1,049 males [48.0%], average age 42.3 ± 13.1 years). The age distribution of the study population is similar to that of the city's total population (table 1).

The subjects taking part in the study were in 89.9 % (1937 of 2155) of German, in 4.3 % (92 of 2155) of Turkish, in 0.3 % (6 of 2155) of Austrian and in 5.4 % (117 of 2155) of various other nationalities.

### Prevalence of obesity and overweight, descriptive analysis of physical activity, smoking habits, alcohol consumption, laboratory results and ultrasonography findings

#### Body-Mass Index (BMI)

Classed as overweight or obese in the study population were 59.1% of males (619/1047) and 42.2% of females (476/1129) (table 2). Clearly more males than females were overweight (40.8% vs. 24.9%). In both sexes, the number of persons with normal weight decreased with age. No gender-specific effect was seen with a BMI equal or greater than 30. The proportion with extreme obesity (grade III according to WHO; BMI ≥ 40) is at 1.3% for females and 1.0% for males. Underweight according to the WHO definition was most commonly seen in females aged 18 to 30 years at 9.3%.

#### Physical activity

2,173 study participants rated their own physical fitness on a scale of 0 to 10 points. Self-assessed mean score was 6.5 ± 1.9 (median: 7; range: 0–10). A degree of physical activity sufficient to "break a sweat" carried on for more than two hours per week was reported by 31.2% (678/2173) of study subjects. Data on leisure time physical activity in hours per week are summarized in table 3. Of all adults 38.9% (847/2,173) do not participate in any physical activity. Gender differences occurred in both this group and in the group with low physical activity (0–2 hours/week). The proportion of males grows with increasing intensity of physical activity from 46.2% (391/847) to 68.4% (26/38). Intensity of physical activity in relation to age, revealed that in the youngest age group females were the least physically active. In the older age groups, the proportion of those who do not participate in sports is similar for females and males.

#### Tobacco consumption

Data on tobacco consumption was provided by 2,175 adults (table 4). The percentage of current smokers in the study population was 30.1% (n = 654), former smokers 24.1% (n = 525) and of non-smokers 45.8% (n = 996). Among current smokers, the proportion of males (55.2%, n = 361) is slightly higher than that of females (44.8%, n = 293). In former smokers the proportion is 55.4% (n = 291) males to 44.6% (n = 234) females and in non-smokers 39.1% (n = 389) males to 60.9% (n = 607) females.

Of former and current smokers, 83.9% (979 of 1,167) reported a daily tobacco use of 0–20 cigarettes (table 4). The median duration of tobacco consumption was in the class of > 10–20 years in both former and current smokers. 29.6% (338/1141) of those surveyed reported having smoked for more than 20 years.

Among former and current smokers, the group of older men reported the highest daily tobacco use. The cohort of young women aged 18–30 years revealed the highest proportion of smokers, smoking < 10 cigarettes per day, at 57.4% compared with 44.4% of the corresponding group of males. The number of heavy smokers (> 40 cigarettes per day) is relatively low, standing at 0.6% in females and 3.6% in males.

#### Alcohol

A third of the population sample (33.3%, 707 of 2,120 persons) reported that they infrequently or never drink alcohol. Women (42.7%, 476 of 1,116 females) make up a larger part of this group than do men (23.0%, 231 of 1,004 males). The average amount of alcohol consumed for the total population sample (n = 2099) stood at 11.7 ± 17.7 grams per day (median: 4.5; range: 0–135.9 g/day); for males (n = 995) a median of 10.8 grams/day and for women (n = 1,104) a median of 2.5 grams/day (table 5). 60.6% (n = 1,289) of the study population reported at least some alcohol (women, 56.3%, 625 of 1,109 females; men, 65.1%, 664 of 1,020 males). Daily consumption of alcohol is reported by 230 subjects. In this group, the proportion of men (18.6%, 187 of 1,004 males) is higher than that of women (4.1%, 43 of 1,153 females). A very high level of consumption is reached at 80 grams/day, an amount reported by 1.4% (29 of 2,120) of subjects while the often described critical level of more than 20 grams/day in women is reached by 9.3% (104 of 1,116) of female subjects and a level of more than 40 grams/day in men is reached by 13.2% (133 of 1,004) of male subjects. The most common form of alcohol consumed is beer, followed by wine, must, champagne and liquor.

#### Laboratory testing/Serology

Total cholesterol was elevated in 62% of subjects studied (1,346 of 2,161) according to standard criteria. No gender effect was seen. Elevated triglycerides were seen in a total of 20.5% (407/1989). Here, however, males were overrepresented at 30.6% (290/949) compared with females at 11.3% (117/1040). A similar distribution was observed for hepatic enzymes: the number of males with elevated hepatic enzymes was higher than that of females. The largest difference was observed for GGT levels. While elevated GGT levels were found in 16.1% of subjects in the overall sample, the proportion in males (29.9%) was nearly 10 times as high as that of females (3.2%).

#### Ultrasonography

Ultrasound examination showed in 72.5% of subjects (n = 1,772) normal liver parenchyma. Hepatic steatosis (increased echogenicity of the hepatic parenchyma in comparison to the renal parenchyma and/or increased attenuation within the liver) was identified in 27.4% of subjects (n = 671).

Gallbladder stones were identified in 8 % of participants (4.8% of males and 10.9% of females).

### Multiple logistic regression

In the multiple regression the effect of gender, age, BMI, smoking, physical activity, ALT, total cholesterol, HDL, triglycerides, sugary drinks, vegetarian diet and alcohol consumption on the occurrence of steatosis hepatis as well as the effect of sex, age, BMI, smoking, steatosis hepatis, alcohol consumption, total blood cholesterol, HDL, triglycerides, consumption of sugary drinks, vegetarian diet and ALT on the amount of physical activity per week ( < 2 h vs. ≥ 2 h) were examined.

#### Steatosis hepatis

In the multiple logistic regression model male sex (OR = 1.43; p = 0.046), higher age (OR = 1.05 per year; p < 0.001) and higher BMI (OR = 1.29 per kg/m^2^; p < 0.001) as well as higher total cholesterol levels (OR = 1.23 per mmol/l; p = 0.008), triglycerides (OR = 1.31 per mmol/l; p =< 0.001) and ALT (OR = 1.08 per U/l; p =< 0.001) showed strong associations with steatosis hepatis (table 6). Increasing HDL levels (OR = 0.53; p = 0.005) showed a protective effect, while no association could be seen between smoking, physical activity, alcohol consumption, consumption of sugary drinks or vegetarian diet on the development of fatty liver.

#### Physical activity

Taking leisure time physical activity of less than 2 hours per week as dependent variable a strong association with female sex could be shown. Higher BMI, current and former smoking and no alcohol consumption were also associated with less than 2 hours of physical activity (table 7). Moderate alcohol consumption (0 g to 40 g alcohol per day) was stronger associated with more than 2 hours of physical activity per week than heavy alcohol consumption (more than 40 g alcohol per day). Age, steatosis hepatis, total cholesterol, HDL, triglycerides, ALT, consumption of sugary drinks and vegetarian diet were not strongly associated with leisure time physical activity.

#### Analysis of covariance

In the analysis of covariance the effect of gender, age, BMI, smoking, physical activity (leisure time physical activity and physical activity "to break a sweat"), sugary drinks, vegetarian diet and alcohol consumption on GGT as well as on total blood cholesterol were examined.

#### Gamma-glutamyl transpeptidase

ANCOVA showed strong effects of gender, age, physical activity "to break a sweat", smoking, alcohol consumption and BMI with measured GGT levels (table 8). Leisure time physical activity, consumption of sugary drinks or vegetarian diet did not exert a strong effect on GGT. Men hat 6.86 U/l higher mean GGT levels than women. Mean GGT levels increased with the amount of consumed alcohol per day.

#### Total cholesterol

Total cholesterol levels were strongly influenced by gender, age, alcohol consumption and BMI. No strong association was found with smoking, leisure time physical activity, consumption of sugary drinks or vegetarian diet. Results of ANCOVA are summarized in table 9 as parameter estimates with 95% confidence intervals and corresponding p-values. Women showed slightly higher mean total cholesterol levels than men. Mean cholesterol levels were increasing with increasing consumption of alcohol, higher BMI and older age.

## Discussion

Currently only few studies have been conducted in Germany which make use of ultrasound, laboratory and survey data to assess multiple indicators of health behaviour in a representative sample of an unselected population [10,16]. In fact, the EMIL study is the first population based study conducted in an urban population in southern Germany to utilize the above-mentioned resources. The rate of participation stood at 62.8%, which compares well with other cross-sectional surveys [16-19]. Analysis of data for the total population shows that the sample in the present study seems to be representative of the local population (table 1).

Of the subjects included in our sample, 17.8% (females, 17.3%; males, 18.3%) were obese (BMI ≥ 30) and 32.5% (females, 24.9%; males, 40.8%) were overweight (BMI 25 – < 30). Our data therefore confirm the increasing problem of obesity in Germany. Overweight and obesity is considered the third most important risk factor of attributable burden of disease in high-income countries [20]. Comparing our data to those of the 1998 *German Nutrition Survey (GNHIES) *we see that the percentage of overweight in both, men and women is lower than those reported for the former West Germany (40.8% vs. 49.5% for males; 24.9% vs. 31.4% for females) [19]. The picture is different for the subgroup with obesity. The subgroup of obese women in the EMIL population stands at 17.3%, which is clearly lower than GNHIES data for West Germany, while the rate for males is similar to that reported in the GNHIES study (18.3% vs. 17.7%) [19]. Studies in an urban population in Malmö, Sweden, showed an increase in overweight and obesity over an eight-year period from 33.9% to 45.2% in males and from 19.6% to 29.1% in females [21]. Current BMI values from a study of medical students aged 20–40 years in Greece show an almost identical picture to that presented by our own subgroup of overweight (26.7%; 264 of 987) [22]. Studies focusing on the prophylaxis of nutrition-related diseases show that over 70% of cases of disease can be prevented by a balanced diet and healthy lifestyle [23]. Corresponding programs in health education and promotion of school programs for children and adolescents can contribute to primary prevention and are certainly justified in terms of future cost savings in the healthcare system [2].

The majority of published studies on physical activity are prospective cohort studies showing an association between low physical activity and the risk of coronary heart disease [24]. In a comparable cross-sectional study in northern Germany, regular physical exercise was reported in 27.6% of subjects aged 45–70 years [25]. In our population sample 31.1% of subjects in the 41–65 year age group reported >0–2 hours of physical activity per week, while 18.3% reported >2–4 hours per week and 9.8% reported >4 hours of physical activity per week. In this group 39.4% of males and 42.1% of females, however, reported no physical exercise. A basic problem in assessing parameters related to physical activity are the survey instruments used in each respective study, which can result in significant differences in the reported physical fitness of the populations surveyed [26].

The health consequences of tobacco consumption, especially the corresponding increased incidence of bronchial carcinoma in prolonged tobacco abuse, are well known [27]. Smoking is the most important attributable risk factor to the burden of disease in high-income countries [20]. In the period from 1985 to 2002, the prevalence of active male smokers in Germany decreased from 40.8% to 34.4%, while the prevalence of active female smokers increased in the same period from 26.1% to 30.7% [28]. In our study population, 34.4% of males and 25.7% of females were considered active smokers at the time of the survey. The highest prevalence of active and former smokers in our study population was in the group of young females aged 18–30 years. In this group, fully 57.4% of women were either current or former smokers, whereas this statement applied to only 44.4% of men in the same age group. The percentage of heavy smokers (11–30 cigarettes per day) is, however, nearly twice as high in males as in females. Our data like others show a trend toward equality between males and females in terms of their smoking behaviour [29].

Beside tobacco, alcohol can also be associated with significant health risks. There is a close association between tobacco use and alcohol consumption [29-31]. In an analysis of the prevalence of high-risk alcohol consumption in Germany published in 1996, the authors report high alcohol consumption in 15.0% of persons living in the southwestern German state of Baden-Württemberg and in 21.4% of residents of Bavaria, with a clear north-to-south gradient for Germany as a whole [9]. Defining high-risk alcohol consumption as a daily alcohol intake in excess of 20 grams per day in females and 40 grams per day in males, the prevalence figures for our population sample (9.3% in females; 13.2% in males) are somewhat lower than those published by Meyer et al. for Baden-Württemberg [9]. Compared with data from the MONICA study in Augsburg (western Bavaria), our data on median daily alcohol consumption (10.8 grams for males; 2.5 grams for females) are as well somewhat lower [11]. Assigning subjects to one of three groups of non-drinkers and those with daily alcohol consumption of 0–40 grams and > 40 grams, Luedemann et al. [25] report rates of 34.4% for non-drinkers, 55.3% for moderate drinkers and 10.3% for high-risk consumers of alcohol in a population in northeastern Germany. These data are almost identical to those reported for our population sample, with 33.3% for non-drinkers and 55.4% for moderate drinkers, respectively. Only the prevalence of high-risk alcohol consumption was lower in our population sample at 6.6%. Daily alcohol consumption in excess of 80 grams is reported by 1.4% of residents of Leutkirch. Similar findings were published for the TACOS study, which found that 1.7% of the population studied exhibited a daily alcohol consumption ≥ 90 grams [18].

Alcohol consumption showed a highly significant effect on GGT levels. This influence, which strongly increases with the amount of consumed alcohol, was also shown by Arndt et al. in a study of male construction workers in southern Germany [32]. In this study and in a study of 6846 steel workers in Korea [33] an even stronger influence was seen between GGT levels and BMI. This association was also highly significant in our population sample.

The ultrasound examination detected a gallbladder stone prevalence of 8%, which corresponds to published data for populations in southern Germany [16].

Although a predominance of steatosis hepatis in male subjects is discussed controversially in the literature Clark et al. confirmed our results [34]. Probably the most important risk factor for the development of steatosis hepatis is overweight and obesity. Although comparison of studies is limited by different definitions of overweight and obesity our findings are confirmed by many studies [35-38]. Another controversial issue is the influence of alcohol consumption on the prevalence of steatosis hepatis. While there are studies describing alcohol as a risk factor for steatosis hepatis [37], more and more studies agree with our findings that alcohol has no effect on the development of steatosis hepatis [40-42]. One of the reasons for the differences in this field might be the difficulties in assessing alcohol consumption properly and in a comparable way.

In general a limitation of the conducted study might be a possible selection bias. As it was necessary for the subjects to appear in person in the study location especially persons with illnesses might be underrepresented. Missing values (i. e. missing laboratory parameters) also could have created a selection bias.

## Conclusion

In conclusion, our survey revealed that overall prevalence of preventable risk factors is high. An overall goal to improve public health should address physical activity levels, healthy eating, and alcohol and tobacco consumption. A preventive strategy with a focus on diet and physical activity offers the perspective of improving health in the population.

## Competing interests

The author(s) declare that they have no competing interests.

## Authors' contributions

MMH designed the study, took part in the organization of the study and was drafting the manuscript. SOB was also part of the study organization team, took part in the data acquisition and interpretation. MK was responsible for data analysis and statistics. UB was involved in data acquisition. RAM drafted the manuscript. GS was responsible for the data acquisition of all laboratory analysis. BOB, WK and PK revised the manuscript critically. IP designed the study and was linking the state health authorities with the study group. She took also part in data acquisition and study coordination. WK designed the study, was mainly responsible for the study coordination and data acquisition and wrote the article. All authors read and approved the final version

## Pre-publication history

The pre-publication history for this paper can be accessed here:



## Figures and Tables

**Table 1:** Gender and age distribution of the study population: 2187 adults aged 18 to 65 years in 2002 compared to the municipal population of Leutkirch

Age class (years)	Study population			Population of the city of Leutkirch		
	Females	Males	Total	Females	Males	Total
18 – 30	208 (18.3%)	210 (20.0%)	418 (19.1%)	918 (23.6%)	1010 (25.1%)	1928 (24.3%)
31 – 40	296 (26.0%)	282 (26.9%)	578 (26.4%)	1011 (25.9%)	1076 (26.7%)	2087 (26.3%)
41 – 50	279 (24.5%)	227 (21.6%)	506 (23.1%)	885 (22.7%)	882 (21.9%)	1767 (22.3%)
51 – 65	355 (31.2%)	330 (31.5%)	685 (31.3%)	1084 (27.8%)	1058 (26.3%)	2142 (27.0%)
Total	1138 (100.0%)	1049 (100.0%)	2187 (100.0%)	3898 (100.0%)	4026 (100.0%)	7924 (100.0%)

**Table 2:** Distribution of BMI according to WHO classification [17] in 2187 German adults by gender and age

**Females**						
Age class (years)	Underweight	Normal weight	Overweight	Obesity Degree I	Obesity Degree II and III	Total
	BMI < 18.5	BMI 18.5 – < 25	BMI 25 – < 30	BMI 30 – < 35	BMI ≥ 35	

18 – 30	19 (9.3%)	135 (66.2%)	31 (15.2%)	12 (5.9%)	7 (3.4%)	204 (18.1%)
31 – 40	11 (3.8%)	191 (65.4%)	61 (20.9%)	23 (7.9%)	6 (2.1%)	292 (25.9%)
41 – 50	12 (4.3%)	154 (55.4%)	71 (25.5%)	23 (8.3%)	18 (6.5%)	278 (24.6%)
51 – 65	4 (1.1%)	127 (35.8%)	118 (33.2%)	80 (22.5%)	26 (7.3%)	355 (31.4%)
Total	46 (4.1%)	607 (53.8%)	281 (24.9%)	138 (12.2%)	57 (5.0%)	1129 (100.0%)

**Males**						

Age class (years)	Underweight	Normal weight	Overweight	Obesity Degree I	Obesity Degree II and III	Total
	BMI < 18.5	BMI 18.5 – < 25	BMI 25 – < 30	BMI 30 – < 35	BMI ≥ 35	

18 – 30	4 (1.9%)	136 (64.8%)	49 (23.3%)	15 (7.1%)	6 (2.9%)	210 (20.1%)
31 – 40	2 (0.7%)	117 (41.6%)	123 (43.8%)	31 (11.0%)	8 (2.8%)	281 (26.8%)
41 – 50	1 (0.4%)	79 (34.8%)	107 (47.1%)	31 (13.7%)	9 (4.0%)	227 (21.7%)
51 – 65	1 (0.3%)	88 (26.8%)	148 (45.0%)	69 (21.0%)	23 (7.0%)	329 (31.4%)
Total	8 (0.8%)	420 (40.1%)	427 (40.8%)	146 (13.9%)	46 (4.4%)	1047 (100.0%)

**Table 3:** Hours of exercise per week (physical activity in leisure time) in 2173 German adults by gender and age

**Females**					
Age class (years)	No exercise	0 – 2 hours/week	>2 – 4 hours/week	>4 hours/week	Total

18 – 30	87 (42.0%)	52 (25.1%)	43 (20.8%)	25 (12.1%)	207 (18.3%)
31 – 40	104 (35.3%)	111 (37.6%)	60 (20.3%)	20 (6.8%)	295 (26.1%)
41 – 50	104 (37.6%)	98 (35.4%)	52 (18.8%)	23 (8.3%)	277 (24.5%)
51 – 65	161 (45.6%)	101 (28.6%)	61 (17.3%)	30 (8.5%)	353 (31.2%)
Total	456 (40.3%)	362 (32.0%)	216 (19.1%)	98 (8.7%)	1132 (100.0%)

**Males**					

Age class (years)	No exercise	0 – 2 hours/week	>2 – 4 hours/week	>4 hours/week	Total

18 – 30	55 (26.4%)	45 (21.6%)	47 (22.6%)	61 (29.3%)	208 (20.0%)
31 – 40	118 (42.3%)	72 (25.8%)	52 (18.6%)	37 (13.3%)	279 (26.8%)
41 – 50	80 (35.7%)	73 (32.6%)	43 (19.2%)	28 (12.5%)	224 (21.5%)
51 – 65	138 (41.8%)	96 (29.1%)	61 (18.5%)	35 (10.6%)	330 (31.7%)
Total	391 (37.6%)	286 (27.5%)	203 (19.5%)	161 (15.5%)	1041 (100.0%)

**Table 4:** Number of cigarettes smoked per day in 1167 German adults (current and former smokers) by gender and age

**Females**					
Age class (years)	≤ 10 cig./day	11–20 cig./day	21–30 cig./day	> 31 cig./day	Total

18 – 30	58 (57.4%)	36 (35.6%)	6 (5.9%)	1 (1.0%)	101 (19.3%)
31 – 40	71 (47.7%)	67 (45.0%)	6 (4.0%)	5 (3.4%)	149 (28.5%)
41 – 50	69 (48.9%)	59 (41.8%)	12 (8.5%)	1 (0.7%)	141 (27.0%)
51 – 65	73 (55.3%)	51 (38.6%)	4 (3.0%)	4 (3.0%)	132 (25.2%)
Total	271 (51.8%)	213 (40.7%)	28 (5.4%)	11 (2.1%)	523 (100.0%)

**Males**					

Age class (years)	≤ 10 cig./day	11–20 cig./day	21–30 cig./day	> 31 cig./day	Total

18 – 30	51 (44.4%)	51 (44.4%)	12 (10.4%)	1 (0.9%)	115 (17.9%)
31 – 40	55 (31.1%)	84 (47.5%)	29 (16.4%)	9 (5.1%)	177 (27.5%)
41 – 50	42 (30.4%)	60 (43.5%)	22 (15.9%)	14 (10.1%)	138 (21.4%)
51 – 65	46 (21.5%)	106 (49.5%)	29 (13.6%)	33 (15.4%)	214 (33.2%)
Total	194 (30.1%)	301 (46.7%)	92 (14.3%)	57 (8.9%)	644 (100.0%)

**Table 5:** Alcohol consumption in grams per day in 2120 German adults by gender and age

**Females**							
Age class (years)	Ex-drinkers	Non-drinkers	>0–20 grams alcohol/day	>20–40 grams alcohol/day	>40–80 grams alcohol/day	>80 grams alcohol/day	Total

18 – 30	5 (2.5%)	103 (51.0%)	84 (41.6%)	8 (4.0%)	1 (0.5%)	1 (0.5%)	202 (18.1%)
31 – 40	6 (2.1%)	128 (44.0%)	133 (45.7%)	21 (7.2%)	3 (1.0%)	0 (0.0%)	291 (26.1%)
41 – 50	10 (3.7%)	104 (38.2%)	126 (46.3%)	27 (9.9%)	5 (1.8%)	0 (0.0%)	272 (24.4%)
51 – 65	21 (6.0%)	141 (40.2%)	151 (43.0%)	32 (9.1%)	6 (1.7%)	0 (0.0%)	351 (31.5%)
Total	42 (3.8%)	476 (42.7%)	494 (44.3%)	88 (7.9%)	15 (1.3%)	1 (0.1%)	1116 (100.0%)

**Males**							

Age class (years)	Ex-drinkers	Non-drinkers	>0–20 grams alcohol/day	>20–40 grams alcohol/day	>40–80 grams alcohol/day	>80 grams alcohol/day	Total

18 – 30	9 (4.5%)	62 (31.0%)	80 (40.0%)	26 (13.0%)	14 (7.0%)	9 (4.5%)	200 (19.9%)
31 – 40	7 (2.6%)	75 (27.8%)	102 (37.8%)	49 (18.2%)	28 (10.4%)	9 (3.3%)	270 (26.7%)
41 – 50	9 (4.2%)	37 (17.1%)	99 (45.6%)	44 (20.3%)	25 (11.5%)	3 (1.4%)	217 (21.6%)
51 – 65	22 (6.9%)	57 (18.0%)	126 (39.8%)	67 (21.1%)	38 (12.0%)	7 (2.2%)	317 (31.6%)
Total	47 (4.7%)	231 (23.0%)	407 (40.5%)	186 (18.5%)	105 (10.5%)	28 (2.8%)	1004 (100.0%)

**Table 6:** The risk (Odds ratio) of hepatic steatosis in ultrasonography according to age, gender, anthropometric measures, blood lipids and liver enzymes. A logistic regression analysis of 1767 adults

Variable	Odds ratio	95% Confidence interval	p-value
Sex (male vs. female)	1.43	1.01; 2.05	0.046
Age (years)	1.05*	1.04; 1.07	< 0.001
BMI (kg/m^2^)	1.29*	1.24; 1.34	< 0.001
Total cholesterol (mmol/l)	1.23*	1.06; 1.43	0.008
HDL (mmol/l)	0.53*	0.34; 0.82	0.005
Triglycerides (mmol/l)	1.31*	1.13; 1.51	< 0.001
ALT (U/l)	1.08*	1.06; 1.10	< 0.001

* per unit

**Table 7:** The risk (Odds ratio) of less than 2 hours of leisure time physical activity per week according to gender, anthropometric measures, tobacco and alcohol consumption. A logistic regression analysis of 1771 adults

Variable	Odds ratio	95% Confidence interval	p-value
Sex (male vs. female)	0.77	0.61; 0.98	0.032
BMI (kg/m^2^)	1.04*	1.01; 1.07	0.005
smoking former vs. none	1.02	0.80; 1.31	0.007
smoking current vs. none	1.43	1.13; 1.80	0.007
≤ 20 g alcohol per day vs. no alcohol per day vs. 0	0.65	0.52; 0.81	< 0.001
>20 g alcohol ≤ 40 g per day vs. no alcohol per day	0.57	0.42; 0.79	< 0.001
>40 g alcohol per day vs. no alcohol per day	0.70	0.46; 1.08	< 0.001

* per unit

**Table 8:** Analysis of covariance for Gamma-glutamyl transpeptidase according to age, gender, anthropometric measures and tobacco and alcohol consumption (n = 1983)

Variable	Parameter estimate (U/I)	95% Confidence interval (U/I	p-value
Sex (male vs. female)	6.86	5.42; 8.30	< 0.001
Age (years)	0.14	0.08; 0.20	< 0.001
BMI (kg/m^2^)	0.48	0.34; 0.63	< 0.001
Physical activity "to break a sweat"	2.09	0.08; 3.49	0.004
Smoking current vs. none	2.07	0.01; 2.54	
Smoking current vs. former non	1.63	0.08; 1.72	0.036
alcohol >40 g/day vs. none	13.12	10.29; 15.94	
alcohol >40 g/day vs. ≤ 20 g/day	12.56	9.85; 15.26	< 0.001
alcohol >40 g/day vs. >20 g/day	10.10	7.07; 13.13	
alcohol ≤ 40 g/day			

**Table 9:** Analysis of covariance for total cholesterol according to age, gender, anthropometric measures and alcohol consumption (n = 1983)

Variable	Parameter estimate (mmol/l)	95% Confidence interval (mmol/l)	p-value
Sex (female vs. male)	0.11	0.01; 0.20	< 0.028
Age (years)	0.03	0.03; 0.04	< 0.001
BMI (kg/m^2^)	0.02	0.01; 0.03	0.001
alcohol >40 g/day vs. none	0.20	0.01; 0,38	
alcohol >40 g/day vs. ≤ 20 g/day	0.08	-0.10; 0.26	0.039
alcohol >40 g/day vs. >20 g/day	0.03	-0.17; 0.23	
alcohol ≤ 40 g/day			
